# Metabolic hijackers: how viral proteins redefine host cell landscapes

**DOI:** 10.1128/jvi.00556-25

**Published:** 2026-01-09

**Authors:** Adam Hafner, Rebekah L. Mokry, John G. Purdy, Christiane E. Wobus

**Affiliations:** 1 Department of Microbiology and Immunology, University of Michigan 1259, Ann Arbor, Michigan, USA; 2 Department of Immunobiology, University of Arizona 8041, Tucson, Arizona, USA; 3 BIO5 Institute, University of Arizona 8041, Tucson, Arizona, USA; 4 Cancer Biology Interdisciplinary Program, University of Arizona 8041, Tucson, Arizona, USA; Indiana University Bloomington, Bloomington, Indiana, USA

**Keywords:** cellular metabolism, RNA and DNA virus proteins, glycolysis, glutaminolysis, lipid metabolism

## Abstract

Viruses are metabolic engineers of host cells. As obligate intracellular pathogens, they rely on host cell metabolism for efficient viral replication. The manipulation of host metabolic processes is a strategy shared among diverse virus families to secure the necessary resources for replicating new genomes, building more virus particles, and supporting cell growth and proliferation. Key metabolic pathways targeted by viruses for disruption and manipulation are glycolysis, glutaminolysis, and lipid metabolism. However, the mechanisms behind virus-induced metabolic reprogramming and the viral proteins mediating it remain poorly understood. This review explores how specific viral proteins reshape the metabolic milieu of host cells during viral infections. We also highlight common themes and outline gaps in knowledge to stimulate further investigations into how viral proteins manipulate host metabolism. Such mechanistic insights will deepen our understanding of virus-host interactions and may reveal novel therapeutic targets.

## INTRODUCTION

Viruses have evolved numerous strategies to ensure successful infection and replication within hosts. One of these is the manipulation of host metabolism. As obligate intracellular parasites, viruses are dependent on their hosts for much of the biological machinery needed for replication. Since many viruses are also metabolically inert, they rely on hijacking host metabolic processes to create a more favorable intracellular environment, ensuring a consistent availability of resources required to produce viral progeny (1).

To meet the energy demand necessary for sustaining viral replication and provide building blocks for macromolecule synthesis, viral infections drive the catabolism of key metabolites, such as glucose and glutamine (2). Simultaneously, viruses promote anabolic processes to hijack the host cell’s macromolecule production, aiding in genome replication and viral particle assembly (3). A key metabolic hallmark for several virus infections is the Warburg Effect, which primes host cells for maintaining virus replication (4, 5). This process increases glycolytic activity in cells in the presence of adequate oxygen, generating the necessary reducing equivalents required for macromolecule biosynthesis or supporting cell growth following virus infections (4). Additional host metabolic pathways increased by viruses and dysregulated in cancerous cells include nucleotide and lipid biosynthesis, as well as glutaminolysis (6, 7). These pathways are often usurped to divert the production of lipids, nucleotides, amino acids, and other metabolites toward virus particle construction and genome replication (8). However, it is important to recognize that host metabolism is a web of interconnected pathways. Thus, when one portion of the web is significantly altered, there are repercussions throughout the entire metabolic network.

**Fig 1:**
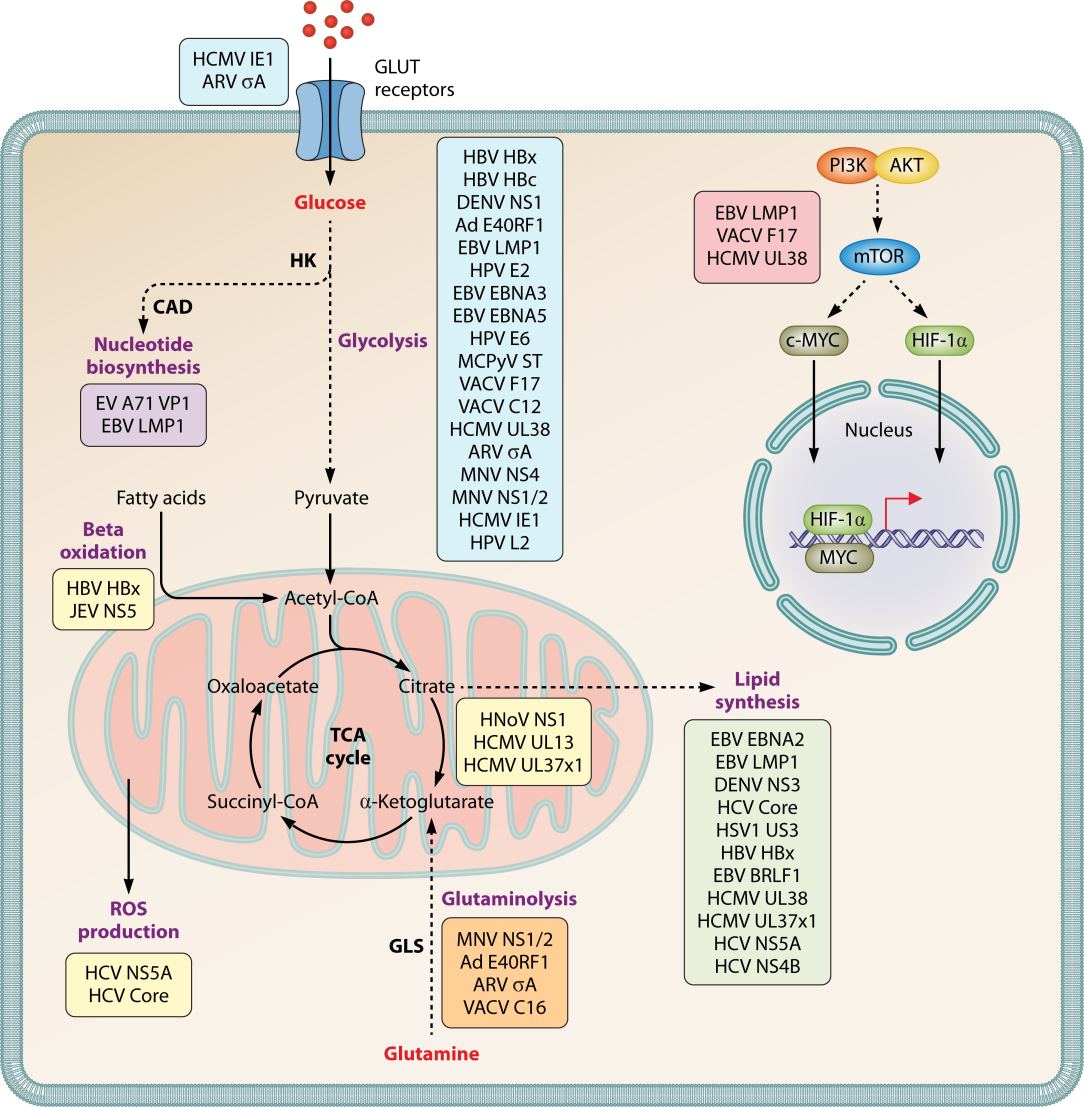
Summary of metabolic pathways altered by viral proteins.

Although metabolic pathways rewired during many virus infections have been known to virologists for decades, the mechanisms behind virus-induced metabolic reprogramming are quite poorly understood. Since viral proteins play a crucial role in engineering the metabolic landscape of infected cells to ensure optimal virus replication, this review will highlight the current knowledge on viral proteins from DNA and RNA virus families that reprogram host metabolic pathways (Fig. 1). Although metabolic reprogramming is essential for all viruses, including plant and insect viruses, we focused this review on viruses that infect mammalian hosts. Our goal is to focus on the known mechanisms of actions of specific viral proteins during the key metabolic pathways of glycolysis, glutaminolysis, and lipid metabolism. We will further discuss key themes that have emerged from these investigations to raise awareness around viral proteins as primary drivers of virus-induced metabolic reprogramming and point out open questions. It is our goal to enhance the field’s understanding of the diverse roles that viral proteins play during infections and stimulate new research in this area to ultimately deepen our understanding of host-pathogen interactions and potentially uncover novel targets for therapeutic intervention.

## GLYCOLYSIS

Glycolysis, the first metabolic pathway discovered, has since become one of the most thoroughly investigated, providing essential insights into cellular energy production and metabolic regulation (9). Glycolysis takes place in the cytosol and begins when glucose enters the cell via a family of glucose transporters (GLUT) (10). During glycolysis, glucose is converted to pyruvate, producing two units of adenosine triphosphate (ATP) and nicotinamide adenine dinucleotide (NADH) per molecule of glucose (11). Pyruvate can then be converted into lactate and exported out of the cell or converted to acetyl-CoA, which enters the tricarboxylic acid (TCA) cycle to produce ATP via the process of oxidative phosphorylation (OXPHOS) or contributes to biomass production, including fatty acid (FA) synthesis (11). Biosynthetic intermediates of glycolysis can also be used in additional pathways such as the pentose phosphate pathway (PPP) for nucleotide biosynthesis, the serine biosynthetic pathway for amino acid production, or lipid synthesis (11). Given the many crucial molecules and metabolites produced during glucose breakdown, it is unsurprising that many viruses actively manipulate these processes during their replicative cycles to yield key resources for successful replication.

Several viral proteins directly influence glycolysis, often leading to its upregulation or, less frequently, its downregulation (Table 1). Other viral proteins indirectly boost glycolytic activity by activating signaling cascades (Table 1). To date, these particular strategies appear to be specific to individual viruses. However, studies between viral proteins from the same virus family are missing, highlighting the need for comparative studies.

**TABLE 1:** Viral proteins known to alter glycolysis

Viral protein	Virus	Pathway	Citation
NS1	Dengue virus	Glycolysis	(12)
NS4	Murine norovirus	Glycolysis	(13)
NS1/2	Murine norovirus	Glycolysis	(13)
Small T antigen	Merkel cell polyomavirus	Glycolysis	(14)
HBc	Hepatitis B virus	Glycolysis	(15)
IE1	Human cytomegalovirus	Glycolysis	(16)
σA	Avian reovirus	Glycolysis	(17)
E4ORF1	Adenovirus	Glycolysis	(18)
E6	Human papillomavirus	Glycolysis	(19, 20)
E2	Human papillomavirus	Glycolysis	(21)
LMP1	Epstein-Barr virus	Glycolysis	(22–25)
EBNA3 and EBNA5	Epstein-Barr virus	Glycolysis	(26)
UL38	Human cytomegalovirus	Glycolysis	(27)
L2	Human papillomavirus	Glycolysis	(28)
HBx	Hepatitis B virus	Glycolysis	(29)
C12, C16, and F17	Vaccinia virus	Glycolysis	(30, 31)

### Viral proteins directly increase glycolysis

One way for viruses to increase metabolic activity and upregulate glycolysis is to directly interact with enzymes in the glycolytic pathway. Alternatively, viral proteins can also increase the expression of metabolic enzymes or glucose transporters, thereby enhancing glycolytic activity. One example of the former is the dengue virus (DENV) non-structural protein NS1, which recruits glyceraldehyde-3-phosphate-dehydrogenase (GAPDH) to its replication centers in the perinuclear region. By directly interacting with GAPDH, NS1 increases enzymatic activity and upregulates glycolysis (12), channeling metabolic fuel directly to the viral replication complex. Another example involves murine norovirus (MNV). MNV-1 increases glycolytic metabolites and glycolytic flux in infected macrophages (13, 32). Its non-structural proteins NS1/2 and NS4 interact with multiple glycolytic enzymes, including hexokinase (HK) 1-3, phosphofructokinase, fructose-bisphosphate aldolase A, GAPDH, and alpha-enolase in the replication complex (13). However, it is currently unknown whether the heightened glycolysis observed during MNV infection is the result of increased activity of one or multiple glycolytic enzymes. This highlights a current unknown in the field: whether reprogramming is achieved through the action of a single protein or the synergistic action of multiple viral proteins. Another knowledge gap in the field is whether viruses encode proteins with redundant abilities to reprogram metabolic pathways.

Several examples of altered glycolytic gene expression have been described. In the case of Merkel cell polyomavirus, infected lung fibroblasts expressing small T antigen show increased expression of glycolytic genes, while simultaneously also downregulating genes involved in OXPHOS (14). This suggests that small T antigen reprograms the cell to favor anaerobic glycolysis, an inefficient means of producing ATP, while providing an efficient source of building blocks required for viral replication (14). Another example is the Hepatitis B core antigen (HBc), which upregulates glycolysis when transfected into HepG2 cells. This upregulation occurs through increased expression of glycolytic enzymes such as phosphoglycerate kinase 1, fructose-bisphosphate aldolase C, and phosphoenolpyruvate carboxykinase, all of which play important roles in enhancing glycolytic activity (15).

Human cytomegalovirus (HCMV) and Avian Reovirus (ARV) provide examples for the upregulation of glucose transporters. HCMV increases glycolysis by inducing the expression of the high-capacity GLUT4 glucose transporter while reducing the more ubiquitous GLUT1 transporter that is normally expressed in fibroblast cells (16). The viral immediate early protein 1 (IE1) is sufficient to decrease GLUT1 expression; however, the viral protein responsible for altering GLUT4 is unknown. The ARV σA protein is one of three viral proteins that comprise the inner capsid (17). In addition to the structural function, it also increases glycolysis through upregulating the expression of GLUT1 (17). Thus, glycolysis is upregulated in ARV infection, similar to HCMV infections, through increased substrate availability.

In summary, viral proteins can directly enhance glycolysis by increasing both the activity and expression of metabolic enzymes and glucose transporters (Table 5). These direct effects ensure a constant supply of energy and building blocks necessary for sustaining viral replication. However, the detailed mechanisms underlying these actions remain largely unknown. Identifying the specific pathways through which viral proteins regulate key receptors, substrate availability, and enzyme expression will advance future research and may uncover common strategies used by diverse viruses.

### Viral proteins indirectly increase glycolysis

Another mechanism by which viral proteins can increase glycolysis is indirect activation of signaling cascades that ultimately result in the alteration to glycolysis, either up or down (Table 5). These alterations can manifest similarly to the direct effects on glycolysis, with an observed increase in the expression of metabolic enzymes and other key molecules involved in glucose breakdown. For example, the ARV σA protein not only increases glycolysis through increased glucose uptake but also increases both mRNA and protein levels of hypoxia-inducible factor-1 alpha (HIF-1α) and cytosolic myc (c-myc) in HeLa cells, a human cervical cancer cell line, and A549 cells, human lung carcinoma cells (17). HIF-1α and c-myc regulate glycolysis through increased expression of HK2, thus resulting in the upregulation of glycolysis during ARV infection (33). In the case of adenovirus (Ad) serotype 5, the gene product of E4ORF1 directly interacts with nuclear myc (N-myc), which promotes its increased binding to glycolytic gene targets, thus increasing the expression and activity of HK2 and phosphofructokinase-1 (PFK1) (18). Thus, activation of transcription factors, which in turn increase glycolytic gene expression, is another effective viral strategy for increasing glycolysis (Fig. 1).

To promote cervical cancer proliferation, several human papillomavirus (HPV) viral proteins increase glycolysis for rapid cell growth and viral replication. The HPV 16 E6 viral protein enhances glycolysis in human cervical cancer cell lines HeLa and SiHa by downregulating the expression of miRNA-34a (19). This downregulation leads to increased expression of HK1 and lactate dehydrogenase A, both important genes in the glycolytic pathway (19). Additionally, E6 was shown to inhibit the association between Von Hippel-Lindau (VHL) and HIF-1α, suggesting that E6 expression promotes glycolysis under hypoxia by allowing HIF-1α to accumulate (20). In the case of HPV 18, the E2 protein increases HIF-1α expression in HaCaT cells, an immortalized human keratinocyte cell line (21). This, in turn, upregulates the transcription of pyruvate dehydrogenase kinase 1 (PDK1) and carbonic anhydrase IX (CAIX). PDK1 inhibits pyruvate dehydrogenase (PDH), which converts pyruvate into acetyl-CoA, thus inhibiting OXPHOS and pushing the infected cells to rely more heavily on glycolysis, whereas CAIX deacidifies cells in response to increased lactate production (21).

Epstein-Barr virus (EBV), an oncogenic human herpesvirus, has multiple mechanisms to indirectly increase glycolysis. Latent membrane protein 1 (LMP1) has been extensively studied due to its role in transforming host cells and is considered the major oncogenic viral protein of EBV (34). Given that EBV transforms host cells, it is not surprising that glycolysis is significantly rewired throughout EBV replication. During nasopharyngeal carcinoma (NPC), LMP1 alters glycolysis by increasing the expression of HK2, thus pushing the cell to favor aerobic glycolysis, a hallmark of oncogenic transformation (22). Specifically, LMP1 stimulates PI3-K/Akt-GSK3beta-FBW7 signaling to upregulate c-myc, which promotes HK2 expression and elevated glycolysis (22). Simultaneously, LMP1 further upregulates glycolysis in NPC cells by decreasing the expression of the homeobox gene cluster C8 (HoxC8) (23). This reduction in HoxC8 expression counteracts the gene cluster’s suppression of glucose consumption, leading to increased HK2 and GLUT-1 receptor expression and lactic acid production (23). Another mechanism by which LMP1 alters glycolysis in NPC cells and a Burkitt lymphoma cell line is by promoting the localization of DNA methyltransferase-1 to the mitochondria. This enzyme inhibits OXPHOS, thereby further promoting aerobic glycolysis (24). Finally, LMP1 also affects glycolysis by increasing the secretion of insulin-like growth factor 1 (IGF-1) and promoting phosphorylation of the IGF1 receptor that activates the mTORC2/AKT signaling cascade. This, in turn, regulates the activity of numerous glycolytic enzymes and the translocation of glucose transporters to the membrane to facilitate increased glucose uptake (25, 35). In addition to LMP1, the EBV nuclear antigens (EBNA) 3 and 5 directly bind to prolyl-hydroxylase 1 and 2, which inhibit HIF-1α hydroxylation and degradation (26). This results in HIF-1α stabilization and translocation to the nucleus to rewire glycolysis, further promoting optimal EBV replication (26). Another herpesvirus protein, HCMV UL38, increases glucose consumption and glycolytic production of lactate by inhibiting the tuberous sclerosis complex 2 (TSC2) (27). In this case, the mechanism used by TSC2 to regulate glycolysis is unidentified, but it is known to be independent of mTOR.

Collectively, recent investigations have shown that viral proteins can indirectly enhance glycolysis by activating cellular master regulators that initiate signaling cascades, by downregulating molecules that negatively modulate glycolysis, and by directly interacting with enzymes that positively influence this metabolic pathway (Table 5). At least one virus, to date, EBV, is using a combination of these mechanisms, pointing to the critical importance of increased glycolysis during pathogenesis. However, as with viral proteins that directly increase glycolysis, the detailed mechanisms of action remain largely unknown.

### Viral proteins that decrease glucose breakdown

As outlined above, glucose catabolism not only provides numerous key nutrients for the health of host cells but also provides viruses with key building blocks for replication and particle assembly. Therefore, most viral proteins identified to date increase glucose uptake and/or glycolysis. However, some viral proteins also decrease glucose breakdown. For example, treatment of SiHa and CaSki cells with purified recombinant HPV-16 protein L2 significantly reduces glucose uptake, lactate production, and oxygen consumption rate, resulting in decreased glycolysis (28). Another example is HBV, since the expression of the HBVx protein (HBx) during infection of the hepatocellular carcinoma cell lines HepG2 and SK-HEP-1 decreases the concentrations of glucose and glucose-6-phosphate, resulting in reduced glycolytic activity (29). Vaccinia virus (VACV) uses its C16 protein to stabilize HIF1α during hypoxic conditions by directly binding to oxygen-sensing enzyme prolyl-hydroxylase domain-containing protein-2 (PHD2) (30). Despite VACV C16 stabilization of HIF-1α, which has been shown to increase glycolysis in other virus infections, VACV decreases glycolysis. The VACV protein F17 localizes to mitochondria and dysregulates mTOR activity to reduce glycolysis and suppress interferon-stimulated gene expression (31).

The reasons for some viruses to favor a decrease in glucose breakdown may be the ability of certain viral replicative cycles to shift their metabolic requirements to additional pathways, such as nucleotide biosynthesis via the PPP, to sustain replication (36). In addition, some viruses may activate alternative pathways to bypass the host’s antiviral defenses, which often target glycolysis (37). This is done to subvert innate immunity as glycolysis supplies the rapid energy and metabolites necessary for immune cell functions (38). Additionally, metabolites produced from glycolysis, such as lactate, can act as a signaling molecule, influencing immune pathways and promoting inflammatory responses (38). This makes the downregulation of glycolysis an attractive option for innate immune evasion for certain viruses.

## GLUTAMINOLYSIS

Glutaminolysis is the process of glutamine breakdown, which begins when glutamine enters the cytosol through transporters such as the alanine-serine-cysteine transporter 2 (ASCT2; encoded by the gene SLC1A5) (39). Once inside, glutamine undergoes two deaminase reactions to produce first glutamate and then alpha-ketoglutarate. These reactions are catalyzed by glutaminase (GLS) and glutamate dehydrogenase (GDH), respectively (39). As one of the primary carbon sources utilized by mammalian cells, glutamine is essential for life (7). Its catabolism provides a nitrogen source to fuel amino acid and nucleotide biosynthesis. Glutaminolysis also supports anaplerosis, the process by which the TCA cycle can be replenished with metabolite intermediates, including those synthesized during glutaminolysis, thus ensuring continuous energy production via OXPHOS (7). Given the importance of glutaminolysis and the essential products synthesized from glutamine breakdown, it is not surprising that viruses have adapted strategies to rewire and hijack this pathway (Table 2).

**TABLE 2:** Viral proteins known to alter glutaminolysis

Viral protein	Virus	Pathway	Citation
NS1/2	Murine norovirus	Glutaminolysis	(32)
E4ORF1	Adenovirus	Glutaminolysis	(40)
σA	Avian reovirus	Glutaminolysis	(41)
C16	Vaccinia virus	Glutaminolysis	(42)

Recent investigations have identified several viral proteins as drivers for virus-induced upregulation of glutaminolysis. For example, macrophages infected with MNV exhibit significantly upregulated glutaminolysis compared to uninfected controls (32). Mechanistic investigations have revealed that the non-structural protein NS1/2 upregulates glutaminolysis through increases in GLS enzymatic activity. In addition to the ability to increase glycolysis, the Ad5 E4ORF1 gene product also increases glutaminolysis during its replicative cycle by directly binding to N-myc, which, in turn, increases the expression of GLS and ASCT2 (40). In the case of ARV, the capsid protein σA not only upregulates glycolysis but also glutaminolysis by activating c-myc, which increases the expression of GLS during ARV infection (41).

Collectively, these viruses use both non-structural or structural proteins interacting with key host enzymes to directly or indirectly upregulate glutaminolysis (Table 5). Interestingly, in the case of Ad and ARV, the same viral proteins E4ORF1 and σA upregulate glutaminolysis and glycolysis (as mentioned in the previous section). MNV also targets both pathways. Although MNV NS1/2 increases glutaminolysis, it also interacts with glycolytic enzymes (13, 32). However, it is currently unknown if NS1/2 also increases glycolytic flux during MNV infection. VACV protein C16, which was shown to stabilize HIF-1α, may be involved in the reprogramming of glutamine metabolism during VACV infection (42). Infection with a virus lacking C16 results in decreased glutamine metabolites, glutamate, 2-hydroxyglutarate (2-HG), and glutathione, compared to WT VACV. However, levels of TCA cycle intermediates were similar in both infection conditions (42). Based on these results, the authors speculate that C16 may act to increase reductive carboxylation during infection (42). Whether other viral proteins known to regulate glycolysis (as mentioned in previous sections) can also alter glutaminolysis is currently unknown.

## LIPID METABOLISM

Lipids are a broad group of chemically and structurally diverse compounds. The complexity of lipid structure is matched by its diverse functions in the cells. Lipids form cellular membranes and function as signaling mediators. They are used for energy storage, precursors for hormones, and as post-translational protein modifications. These biological functions help lipids support the replication of viruses and promote cell growth and proliferation of cells infected with an oncogenic virus. Lipid synthesis is regulated by a series of cellular factors in response to metabolic demand. Metabolites synthesized through other pathways, such as glycolysis and glutaminolysis, are used for lipid synthesis. Acetyl-CoA generated from citrate or acetate is used for FA and sterol synthesis. Acylglycerol lipids have a glycerol backbone derived from dihydroxyacetone phosphate (DHAP) that is redirected from glycolysis to lipid synthesis. ATP and NADPH provide energy and reducing power for lipid synthesis. Both enveloped and non-enveloped viruses modulate lipid metabolism (Table 3). Lipids likely play roles in energy maintenance, viral assembly, envelopment for enveloped and quasi-enveloped viruses, viral egress, and viral entry. However, the function of many lipids is not yet fully elucidated in infected cells. In the following section, we will discuss relevant lipid synthesis pathways and describe the viral proteins that modulate these processes.

**TABLE 3:** Viral proteins known to alter lipid metabolism

Viral protein	Virus	Pathway	Citation
UL38	Human cytomegalovirus	Lipid metabolism	(43, 44)
UL37x1/vMIA	Human cytomegalovirus	Lipid metabolism	(45–47)
US3	Herpes simplex virus	Lipid metabolism	(48)
LMP1	Epstein-Barr virus	Lipid metabolism	(49, 50)
EBNA2	Epstein-Barr virus	Lipid metabolism	(51, 52)
BRLF1	Epstein-Barr virus	Lipid metabolism	(53)
NS3	Dengue virus	Lipid metabolism	(54)
NS5B	Hepatitis C virus	Lipid metabolism	(55)
NS4A	Hepatitis C virus	Lipid metabolism	(56)
HCV Core Protein	Hepatitis C virus	Lipid metabolism	(57)
HBx	Hepatitis B virus	Lipid metabolism	(58–60)
HBx	Hepatitis B virus	β-oxidation	(61)
NS5	Japanese encephalitis virus	β-oxidation	(62)

### Lipogenesis and its regulation

Lipid synthesis is largely regulated by sterol responsive element-binding proteins (SREBPs), transcription factors that regulate the expression of lipogenic genes. Three SREBP isoforms coordinate lipid metabolism. In general, SREBP1a is a global regulator of lipid synthesis, SREBP1c is involved in FA synthesis and storage, and SREBP2 is involved in cholesterol regulation (63). SREBPs form complexes in the ER with SREBP cleavage-activating protein (SCAP). Another protein called insulin-induced gene 1 protein (INSIG) binds the SREBP-SCAP complexes when the need for lipid synthesis is low. Upon receiving an activating signal, INSIG releases the SREBP-SCAP complex, allowing it to translocate to the Golgi. In the Golgi, SREBPs are sequentially cleaved by S1P and S2P proteases to their mature form that can move to the nucleus. Mature SREBPs are transcription factors for lipogenic genes containing sterol regulatory elements (SRE).

Some SRE-containing genes regulate sterol synthesis, including cholesterol. Sterol synthesis occurs in four stages, beginning with acetyl-CoA. In the first stage, three acetyl-CoA units combine to form mevalonate. The rate-limiting step of sterol synthesis occurs in this stage with the formation of mevalonate from HMG-CoA by HMG-CoA reductase (HMGCR). Mevalonate is then converted to activated isoprene units by a series of reactions, followed by the formation of a 30-carbon squalene from six molecules of 5-carbon isoprene. Squalene is converted to a four-ring steroid nucleus structure. The final steps in the pathway generate cholesterol. Cholesterol can be further metabolized to cholesteryl ester (i.e., cholesterol with a fatty acyl tail) or bile acids. It also serves as the precursor for steroid hormones. NADPH provides the reducing power for two steps in sterol synthesis, and ATP is required for the conversion of mevalonate to activated isoprenes.

Other SRE-containing genes regulate FA synthesis, such as acetyl-CoA carboxylase 1 (ACC1) and fatty acid synthase (FASN). Like cholesterol, FAs are synthesized from acetyl-CoA. Cytosolic acetyl-CoA is produced from citrate via ATP citrate lyase (ACLY) or from acetate via acetyl-CoA synthetase short-chain family member 2 (ACSS2). The first and rate-limiting step in FA synthesis is the formation of malonyl-CoA from bicarbonate and acetyl-CoA via the enzyme ACC1. FASN uses malonyl-CoA to extend the fatty acyl hydrocarbon chain. The primary product of FASN is palmitate, a saturated FA with a chain length of 16 carbons (C16); however, it can also produce C14 and C18. The newly produced FAs have several fates, including utilization as a post-translational protein modification (e.g., palmitoylation) or attachment to a lipid as a fatty acyl tail. FAs can be further processed by FA elongases (ELOVLs) that extend the tail or stearoyl-CoA desaturases (SCDs) that catalyze the formation of carbon-carbon double bonds. Saturated FAs have no double bonds, whereas polyunsaturated FAs have two or more. The number and placement of the double bonds vary but play important roles in defining the function of the FA. Most FAs have between zero and six double bonds. NADPH provides the reducing power necessary for FA synthesis and elongation, while ATP provides the energy.

Molecules containing a glycerol backbone and one to three FA tails are a class of lipids called acylglycerol lipids and include phospholipids and triacylglycerol (TAG). Phospholipid classes are designated by their heads. The most basic phospholipid is phosphatidic acid (PA), which has a phosphate group and no additional functional group. Other phospholipids have a metabolite-modified phosphate group: choline for phosphatidylcholine (PC), ethanolamine for phosphatidylethanolamine (PE), glycerol for phosphatidylglycerol (PG), serine for phosphatidylserine (PS), and inositol for phosphatidylinositol (PI). The tails of phospholipids, TAGs, and the closely related diacylglycerols (DAGs) vary in length and number, plus the placement of carbon double bonds. Synthesis of acylglycerol lipids starts with the formation of glycolysis-derived DHAP, which is converted to glycerol 3-phosphate. Fatty acids are attached through esterification to the hydroxyl positions of the glycerol 3-phosphate to generate PA. DAG is generated from PA and serves as a hub for the synthesis of PC, PE, PS, PG, PI, and TAG. Phospholipids can also be remodeled through the activity of phospholipases and lysophospholipid acyltransferases, which remove and replace FA tails, respectively.

Several herpesviruses regulate ACC1 or FASN directly through activities of viral proteins, thereby altering FA synthesis. HCMV increases ACC1 expression and FA synthesis (64). ACC1 induction requires viral gene expression but not viral DNA replication, indicating that an immediate-early or early viral gene is responsible, although this protein has not been identified (64). HCMV UL38 increases FA synthesis and elongation through mTOR and mTOR-independent routes, potentially by triggering SREBP maturation (43, 44). UL38 targeting of the TSC2, a tumor-suppressor gene, supports its mTOR-independent metabolic reprogramming (44). HCMV UL38 and UL37x1 (also called viral mitochondria-localized inhibitor of apoptosis; vMIA) proteins promote the level of FA elongase 7 (ELOVL7) and synthesis of FAs (43, 45). UL37x1 supports lipid synthesis in HCMV infection at several levels. UL37x1 helps increase PKR-like endoplasmic reticulum kinase (PERK)-dependent lipid synthesis (45–47). Although PERK’s mechanism of action in regulating lipid metabolism is poorly understood, the study of HCMV revealed that it controls the levels of certain ELOVLs (45–47). Additional HCMV genes may be involved in reprogramming lipid metabolism, and a recent study reported that HCMV specifically induces *de novo* PC synthesis through the expression of an unidentified immediate-early or early gene (65). Interestingly, herpes simplex virus 1 (HSV1) also increases the synthesis of lipids containing choline, with the viral kinase US3 acting to suppress synthesis to maintain membrane integrity (48).

EBV encodes several proteins that regulate lipid synthesis by targeting SREBPs, FASN, and other lipogenic proteins. In addition to driving glycolysis during EBV infection, LMP1 also regulates lipid metabolism. Ectopic expression of LMP1 in NPC increases the expression and maturation of SREBP1 in an mTOR-dependent manner, leading to more FASN expression (49). Complementary experiments in EBV-induced immortalization of B-cells or ectopic LMP1 expression in Burkitt’s lymphoma cell lines show similar results with increased FASN and FAs levels (50). Moreover, LMP1 increases activity of the deubiquitinating enzyme USP2a to stabilize FASN levels (50). EBV nuclear EBNA2 protein is a major transactivator of latent gene expression while also regulating cellular genes. EBNA2 also increases SREBP1 maturation and FASN levels (51). Infection of the human lymphoma cell line, HBL, with EBV or EBNA2-encoding adenovirus increases SREBP1 and FASN levels and lipogenesis (51). Loss of EBNA2 causes a decrease in SREBP1 and FASN and reduces lipogenesis (51). More recently, EBNA2 was shown to increase SCD1 and FA desaturase 2 (FADS2) levels to maintain a balance of saturated to unsaturated FA and support B-cell proliferation (52). During EBV lytic replication, immediate early protein BRLF1, a transcriptional activator, increases FASN levels through p38 kinase activity, and inhibition of FASN results in decreased lytic replication; however, it is unclear if induction of FASN also increases FA synthesis (53). In primary B cells, EBNA2 increases the sterol metabolite geranylgeranyl pyrophosphate (GGPP), which modifies and activates Rab proteins to support B-cell survival and proliferation (66). To do so, EBNA2 binds to the promoters of SREBP2, ACC1, and HMGCR to induce their expression. Induction of HMGCR increases sterol synthesis and GGPP production. In EBV infection models, statin inhibition of sterol synthesis results in decreased cell proliferation and increased cell death. EBNA2 supports mitochondrial remodeling by inducing enzymes involved in cardiolipin synthesis, an acylglycerol lipid important for mitochondria structure (67).

Flaviviruses also regulate lipogenesis through viral protein activity. DENV NS3 protein and HCV NS5B protein both interact with FASN during infection (54, 55). For DENV infection, FASN relocalization is postulated to support initiation and expansion of viral replication sites (54). In DENV-infected cells, increased FASN levels are correlated with increased FA synthesis (54). During HCV infection, FASN may retain and modulate the role of NS5B as an RNA-dependent RNA polymerase at replication sites. Inhibition of FASN reduces both DENV and HCV replication, indicating that FASN is required for successful replication (54, 55). HCV proteins, NS4B and NS5A, and core proteins increase the expression and maturation of SREBP1, which may be mediated by PI3K/AKT signaling (56, 57). HCV core protein also increases the transcriptional activity of peroxisome proliferator-activated receptor gamma (PPARγ), another transcription factor that regulates lipid metabolism. NS4B regulation of SREBP1 correlates with FAS, SCD, and ACC1 induction, and core protein modulation of SREBP1 or PPARγ transcriptional activity correlates with increased FASN and ACC1 (56–58). Moreover, HCV infection or ectopic expression of NS4B results in the accumulation of lipids (56). NS4B also increases the expression and maturation of SREBP2, which correlates with the induction of HMGCR, although increased sterol synthesis was not shown (56).

Hepatitis B virus (HBV) activates the transcriptional activity of SREBP1, PPARγ, and another transcription factor, C/EBPα, via HBx (59). HBx activates SREBP1 through the PI3K/AKT signaling pathway; both SREBP1 and PPARγ-regulated lipogenic genes, such as FASN, ACC1, and SCD, are increased. Moreover, HBx transgenic mice have enlarged livers that accumulate lipids, indicating that HBx induces lipid storage via the regulation of SREBP1 and PPARγ transcriptional activity. A study in HepG2 cells demonstrated that HBx induces fatty acid binding protein 1 (FABP1), which is involved in FA transport and synthesis, via transcriptional activity of PPARγ, C/EBPα, and another transcription factor, HNF3β (60). Again, HBx transgenic mice and HepG2 HBx-expressing cells show increased FABP1 levels correlated with increased lipid accumulation. While these studies show HBx activation of lipogenic genes, we still lack a complete understanding of how the activity in the various lipid metabolism pathways is altered. HBV further highlights that a common theme in viral regulation of lipid metabolism is the activation of SREBPs to induce lipogenic genes. However, the specific mechanisms by which viral proteins modulate SREBP activation are largely unknown and require further study.

### Oxidation of FA

The tails of lipids are an efficient form of energy storage for cells. The generation of energy from lipids primarily occurs in mitochondria through a process known as β-oxidation, by which FAs are oxidized back to acetyl-CoA units. First, FAs are released from lipids by lipases, converted to fatty acyl-CoA by fatty acyl-CoA synthetases, and then converted to fatty acyl-carnitine to allow entrance into the mitochondrial matrix through the carnitine cotransporter. Fatty acyl-carnitine is then converted back to fatty acyl-CoA and is ready for β-oxidation. This process is carried out in four steps: dehydrogenation, hydration, additional dehydrogenation, and thiolysis. The last three steps are catalyzed by the heterotrimeric protein mitochondrial trifunctional protein (MTP). The final thiolysis step of β-oxidation yields acetyl-CoA and the remaining fatty acyl-CoA reduced by two carbons. The four-step process is repeated until the fatty acyl-CoA is oxidized to the last acetyl-CoA. Acetyl-CoA can feed into the TCA cycle to generate additional energy.

To date, virus reprogramming of β-oxidation has been studied less than their reprogramming of lipogenesis (Table 3). The HBV protein HBx induces β-oxidation but only during glucose deprivation to promote cell survival in liver cancer cells (61). HBx activates β-oxidation by triggering the release of cytosolic calcium, which activates calcium/calmodulin-dependent protein kinases (CaMKKs), leading to the phosphorylation of AMPK. Authors correlate increased AMPK phosphorylation with activation of β-oxidation. Overexpression of HBx in mice enhances tumorigenesis, and inhibition of β-oxidation reduces this effect. In contrast to this activity, the flavivirus Japanese encephalitis virus (JEV) inhibits β-oxidation (62). During JEV infection, the viral NS5 protein binds two subunits of the MTP complex, HADHα and HADHβ, to reduce β-oxidation. Preventing this interaction by mutating the NS5-binding site decreased its ability to suppress β-oxidation. JEV infection with a virus that has mutated NS5 did not impact virus replication in cell culture but was less virulent in mouse models of JEV infection. The authors correlated reduced virulence with a reduction in cytokine production resulting from decreased ROS.

## ADDITIONAL METABOLIC PATHWAYS

In addition to glycolysis, glutaminolysis, and lipid metabolism, viruses also hijack and rewire additional pathways involved in host metabolism to obtain resources required for optimal progeny production (Fig. 1). One key pathway targeted is the PPP, which is responsible for generating nucleotides (68). The PPP produces NADPH, an electron carrier essential for anabolic reactions, and ribose-5-phosphate, the sugar backbone necessary for the synthesis of nucleotides and nucleic acids (60). The PPP runs parallel to glycolysis, and certain PPP intermediates can be shunted into the glycolytic pathway, making it another attractive target for viruses to manipulate during their replicative cycles (Table 4). This is exemplified during enterovirus (EV) A71 infection. During EV-A71 infection of Vero cells, increased pyrimidine synthesis is observed (69). Mechanistically, the major capsid protein VP1 directly interacts with the host enzyme complex carbamoyl-phosphate synthetase 2, aspartate transcarbamoylase, and dihydroorotase (CAD), which catalyzes the first three steps of pyrimidine biosynthesis, to increase its enzymatic activity (69). Another example is the EBV protein LMP1 that increases purine synthesis, which is necessary to support cell survival and growth of EBV-transformed cells (70).

**TABLE 4:** Viral proteins known to alter alternative metabolic pathways

Viral protein	Virus	Pathway	Citation
VP1	Enterovirus A71	PPP	(69)
LMP1	Epstein-Barr virus	PPP	(70)
NS1	Human norovirus	TCA cycle	(71)
UL37x1/vMIA	Human cytomegalovirus	Cellular respiration	(57–62, 68–73)
UL13	Human cytomegalovirus	Cellular respiration	(74)
EBNA2	Epstein-Barr virus	One-carbon metabolism	(75)
HCV Core Protein	Hepatitis C virus	ROS production	(76, 77)
NS5A	Hepatitis C virus	ROS production	(78)

An additional pathway targeted by viruses is the TCA cycle to satisfy the massive energy requirement of viral infections (Table 4). The TCA cycle occurs in the mitochondria when pyruvate produced from glycolysis is oxidized into acetyl-CoA, which is utilized to generate energy via a series of redox reactions (11). Human norovirus (HNoV) infects primary human B cells *in vitro* (71). When primary blood-derived human B cells are treated with recombinant HNoV NS1, B cells are activated, and the production of metabolites in the TCA cycle is strongly induced (71). However, whether NS1 can also mediate the upregulation of the TCA cycle during HNoV infection remains unknown.

Mitochondria can also be targeted by viral proteins to support virus production (Table 4). HCMV UL37x1 is sufficient to disrupt mitochondrial structure and supports increased cellular respiration during infection (72, 73). UL37x1 also interacts with HCMV protein UL13 in the mitochondria, although the significance of this interaction is unknown (74). On its own, UL13 localizes to the mitochondria and interacts with several mitochondrial proteins. UL13 alters the structure of the cristae and supports increased cellular respiration during infection (74).

EBV targets a series of metabolic pathways called one-carbon metabolism, which provides one-carbon units for the synthesis of metabolites (75) (Table 4). EBV upregulates one-carbon metabolism during infection through direct binding of EBNA2 to the promoter and enhancer regions of MTHFD2, a central enzyme for one-carbon metabolism, to induce expression. In turn, MTHFD2 mediates one-carbon metabolism that contributes to nucleotide synthesis, glutathione synthesis, and increased NADPH/NADP^+^ ratios during EBV infection.

Another notable alteration in virus-infected cells is the production of reactive oxygen species (ROS). While normal ROS levels can support the host by helping to mount a proper immune response against invading pathogens, excessive ROS production during infections can lead to oxidative stress and cellular damage (79). Thus, while ROS plays a vital role in immune defenses, its dysregulation during viral infections may also contribute to increased pathogenesis and viral replication (Table 4). This is observed during HCV infection, which is characterized by chronic oxidative stress resulting from increased ROS production (76, 79). The HCV core protein mediates the elevated production of ROS in a calcium-dependent manner. HCV core protein expression increases mitochondrial calcium uptake through increased uniporter expression (77). The increased uptake of calcium in the mitochondria results in significant ROS production and mitochondrial permeability transition. This activity also disrupts the electron transport chain, leading to decreased energy production and mitochondrial damage (77, 80). However, the increased ROS production may be advantageous to HCV replication since this activity can alter apoptotic signals that work to prevent apoptosis. HCV NS5A has also been found to increase ROS production (78). NS5A increases mitochondrial ROS production by decreasing FOXO1 phosphorylation and nuclear accumulation. This ultimately leads to the upregulation of phosphoenolpyruvate carboxykinase (PEPCK) and glucose-6-phosphate (G6P) (78). Increased expression of these genes enhances glucose production through hepatic gluconeogenesis, thereby promoting increased HCV replication (78).

In summary, while multiple other metabolic pathways besides glycolysis, glutaminolysis, and lipid metabolism, such as PPP and ROS biosynthesis, are needed for cellular homeostasis and to sustain viral replication, few viral proteins that alter them have been identified to date. However, given the integral dependencies of viruses on the plastic metabolic network, many more interactions are likely to be identified in the future.

## DISCUSSION/FUTURE DIRECTIONS/GAPS

This review highlights the current knowledge on viral proteins from both DNA and RNA virus families, along with their known mechanisms of action in reprogramming host metabolic pathways (Table 5). As obligate intracellular parasites, viruses critically rely on the metabolic products of their hosts to ensure successful reproduction (27). Viral proteins act as agents of metabolic rewiring, creating a more favorable intracellular environment to promote optimal replication. To achieve this, they target important metabolic pathways, including glycolysis, glutaminolysis, lipid metabolism, and additional pathways like ROS synthesis and energy production via the TCA cycle (29) (Fig. 1). Numerous pathways are often targeted because host metabolism is a flexible, interconnected web (81). Any alteration to one section of the web can have consequences for the entire network with viral proteins serving as the architects working to alter specific pathways to ensure the optimal intracellular conditions. This is exemplified when viruses push host cells toward aerobic glycolysis during infections, which generates substrates for nucleotide biosynthesis and also creates a greater dependency on glutaminolysis for sustained energy production (39). While the field of viral metabolism is in its infancy, research thus far has focused on central carbon and lipid metabolism. However, it is also important to investigate if viral proteins specifically alter lesser-studied pathways, such as non-essential or non-proteinogenic amino acid metabolism.

**TABLE 5:** Mechanism of action of viral proteins

Mechanism of action	Metabolic pathway	Viral protein	Virus	Citation	Notes
Directly targets metabolic enzyme	Glycolysis	NS1	Dengue virus	(12)	Increases HK activity
Directly targets metabolic enzyme	Glycolysis	NS4	Murine norovirus	(13)	Recruits glycolytic enzymes to replicase complex
Directly targets metabolic enzyme	Glycolysis	NS1/2	Murine norovirus	(13)	Recruits glycolytic enzymes to replicase complex
Directly targets metabolic enzyme	Glutaminolysis	NS1/2	Murine norovirus	(32)	Increases GLS activity
Directly targets metabolic enzyme	Fatty acid synthesis	NS3	Dengue virus	(54)	Interacts with FASN
Directly targets metabolic enzyme	Fatty acid synthesis	NS3	Hepatitis C virus	(54)	Interacts with FASN
Directly targets metabolic enzyme	Fatty acid synthesis	NS5B	Hepatitis C virus	(55)	Interacts with FASN
Directly targets metabolic enzyme	β-oxidation	NS5	Japanese encephalitis virus	(62)	Binds to subunits of the mitochondrial trifunctional protein
Directly targets metabolic enzyme	Pentose phosphate pathway	VP1	Enterovirus A71	(69)	VP1 binds to CAD in PPP
Directly targets metabolic enzyme	One-carbon metabolism	EBNA2	Epstein-Barr virus	(75)	Directly binds MTHFD2 promoter to induce expression
Alters metabolic gene expression	Glycolysis	Small T antigen	Merkel cell polyomavirus	(14)	Increases glycolytic gene expression
Alters metabolic gene expression	Glycolysis	HBc	Hepatitis B virus	(15)	Increases glycolytic gene expression
Alters metabolic gene expression	Glycolysis	IE1	Human cytomegalovirus	(16)	Decreases GLUT1 expression
Alters metabolic gene expression	Glycolysis	σA	Avian reovirus	(17)	Increases GLUT1 expression
Alters metabolic gene expression	Fatty acid synthesis	LMP1	Epstein-Barr virus	(50)	Increases FASN levels
Alters metabolic gene expression	Fatty acid synthesis	BRLF1	Epstein-Barr virus	(53)	Increases FASN levels
Alters metabolic gene expression	Fatty acid synthesis	EBNA2	Epstein-Barr virus	(66)	Induces the expression of ACC1/HMGCR
Suppresses negative metabolic regulators	Glycolysis	E6	Human papillomavirus	(19)	Decreases miRNA-34a expression
Suppresses negative metabolic regulators	Glycolysis	LMP1	Epstein-Barr virus	(23)	Decreases HoxC8 expression
Suppresses negative metabolic regulators	Glycolysis	EBNA3 & EBNA5	Epstein-Barr virus	(26)	Binds to prolyl-hydroxylase 1 and 2
Suppresses negative metabolic regulators	Fatty acid synthesis	UL38	Human cytomegalovirus	(44)	Targets TSC2 to increase FA synthesis
Suppresses negative metabolic regulators	ROS production	NS5A	Hepatitis C virus	(78)	Decreases FOXO1 phosphorylation
Targets metabolic regulatory transcription factors	Glycolysis	σA	Avian reovirus	(17)	Increases HIF1a/c-myc expression
Targets metabolic regulatory transcription factors	Glycolysis	E4ORF1	Adenovirus	(18)	Interacts with N-myc
Targets metabolic regulatory transcription factors	Glycolysis	E6	Human papillomavirus	(20)	Allows HIF1a to accumulate
Targets metabolic regulatory transcription factors	Glycolysis	E2	Human papillomavirus	(21)	Increases HIF1a expression
Targets metabolic regulatory transcription factors	Glycolysis	LMP1	Epstein-Barr virus	(22)	Activates PI3-K/Akt-GSK3beta-FBW7 signaling to upregulate c-myc
Targets metabolic regulatory transcription factors	Glycolysis	C16	Vaccinia virus	(30)	Stabilizes HIFa expression
Targets metabolic regulatory transcription factors	Glutaminolysis	E4ORF1	Adenovirus	(40)	Binds/activates to N-myc
Targets metabolic regulatory transcription factors	Glutaminolysis	σA	Avian reovirus	(41)	Activates c-myc
Targets metabolic regulatory transcription factors	Fatty acid synthesis	LMP1	Epstein-Barr virus	(49)	Increases expression and maturation of SREBP1 and induces lipogenic gene expression
Targets metabolic regulatory transcription factors	Fatty acid synthesis	EBNA2	Epstein-Barr virus	(51)	Increases SREBP1 maturation and induces lipogenic gene expression
Targets metabolic regulatory transcription factors	Fatty acid synthesis	NS4B	Hepatitis C virus	(55, 56)	Increases expression of SREBP1 and SREBP2 to induce lipogenic genes
Targets metabolic regulatory transcription factors	Fatty acid synthesis	NS5A	Hepatitis C virus	(55, 56)	Increases expression of SREBP1
Targets metabolic regulatory transcription factors	Fatty acid synthesis	Core Protein	Hepatitis C virus	(57, 58)	Increases expression of SREBP1 and PPARgamma to induce lipogenic genes
Targets metabolic regulatory transcription factors	Fatty acid synthesis	HBx	Hepatitis B virus	(59, 60)	Activates SREBP1, PPARgamma, and C/EBPalpha to induce lipogenic genes
Targets master metabolic regulators	Glycolysis	LMP1	Epstein-Barr virus	(25, 35)	Activates mTORC2/AKT
Targets master metabolic regulators	Glycolysis	F17	Vaccinia virus	(31)	Dysregulates mTOR
Targets master metabolic regulators	Fatty acid synthesis	UL37x1	Human cytomegalovirus	(45–47)	Targets PERK
Targets master metabolic regulators	β-oxidation	HBx	Hepatitis B virus	(61)	Increases calcium release, which results in the phosphorylation of AMPK
Unknown	Glycolysis	L2	Human papillomavirus	(28)	Decreases glycolysis
Unknown	Glycolysis	HBx	Hepatitis B virus	(29)	Decreases glycolysis
Unknown	Glutaminolysis	C16	Vaccinia virus	(42)	Increases reductive carboxylation
Unknown	Fatty acid synthesis	US3	Human simplex virus	(48)	Suppresses lipid synthesis
Unknown	Pentose phosphate pathway	LMP1	Epstein-Barr virus	(70)	Increases purine synthesis
Unknown	TCA cycle	NS1	Human norovirus	(71)	Increases TCA cycle metabolites
Unknown	Mitochondrial architecture	UL37x1	Human cytomegalovirus	(72, 73)	Targets mitochondria
Unknown	Mitochondrial architecture	UL13	Human cytomegalovirus	(74)	Targets mitochondria

Investigations thus far have revealed several common themes among the known mechanisms employed by viral proteins (Table 5). The first strategy to upregulate a specific pathway is by enhancing the expression of key metabolic enzymes or increasing their enzymatic activity. Many viruses accomplish this strategy for lipid metabolism by hijacking SREBP regulation to induce enzymes in cholesterol and FA synthesis pathways. Viruses also accomplish this by utilizing their proteins to directly interact with specific metabolic enzymes, which work to increase enzymatic activity. This is observed, for example, with Dengue NS1, which binds with GAPDH to increase its activity and overall glycolytic flux. The second mechanism is for viral proteins to enhance the availability of intracellular substrates to support various metabolic pathways through increased expression of metabolite transporters and is exemplified by HCMV and ARV, which alter GLUT expression to enhance glucose uptake. The third theme is for some viral proteins to activate signaling cascades, indirectly leading to the upregulation or downregulation of specific metabolic pathways. EBV, for example, employs this strategy by activating the PI3-K/Akt/GSK3 signaling pathway to induce c-myc by ultimately inducing HK2 and increasing glycolysis. A fourth strategy utilized is for viral proteins to decrease the expression of negative regulators of certain metabolic pathways. Most viruses use a combination of these strategies for different metabolic needs, giving the virus unfettered access to key resources required to support virus production or cell proliferation. In summary, these shared strategies further illustrate how viral proteins act as metabolic engineers that utilize many diverse approaches aimed at optimizing the metabolic environment for viral replication.

When identifying metabolic modulation by viral proteins, it is important to consider that a protein may be required, but not sufficient, for activating a metabolic pathway. As an example, UL37x1 supports HCMV lipidomic remodeling (36). However, exogenous expression of UL37x1 by itself is unable to induce lipid synthesis similar to infection, indicating other viral factors are necessary for HCMV-mediated shifts in the lipidome. Likewise, expression of UL38 partially recapitulates the metabolic profile of HCMV-infected cells but fails to establish the full metabolic reprogramming caused by infection (27). These observations highlight that expression of a sole viral protein may not mimic the metabolic reprogramming observed in infected cells. In contrast, it is also possible that redundant metabolic activities by viral proteins can mask contributions. It also remains unclear whether different cell types in the infected environment alter the metabolic interplay and the viral proteins that regulate these pathways. These observations serve to remind us that we only have a partial understanding of how viral proteins regulate host metabolism.

While identifying the viral proteins and their impacted metabolic pathway is crucial, many of the mechanisms they use in the rewiring of host metabolism remain unknown. Increased investigations aimed at uncovering these mechanisms will help further identify common strategies employed by various viruses and how viral proteins ensure metabolites and lipids are present when the virus needs them. This information will help uncover if these emerging strategies are conserved within viral families. Increased investigations will also aid in discovering additional viral proteins that alter host metabolism. Such discoveries will help reveal whether viral proteins modulate specific host metabolic pathways or if a single viral protein can alter numerous pathways. As a result, the insights gained will illuminate the replicative strategies of many viruses, enhance our understanding of virus-host interactions, highlight the oncogenic potential of certain viral proteins, and potentially reveal new strategies for therapeutic intervention. This knowledge will help better determine if pharmacological targeting of metabolism provides a therapeutic opportunity for enhancing the activity of direct-acting antivirals for successfully treating viral infections. Throughout the text, we have pointed out some of the many knowledge gaps and summarized them (see Open Questions) to spur further research. Addressing these and related questions in viral metabolomics with established and emerging technologies (e.g., spatial metabolomics and real-time metabolic imaging) will undoubtedly lead to exciting new discoveries in this research area for years to come.

## OPEN QUESTIONS

1. Viruses often target numerous metabolic pathways. Are these metabolic functions of viral proteins conserved within viral families and/or between infected cell types?
2. Are there emerging themes within a viral family or across viral families regarding a given viral protein altering multiple metabolic pathways or one viral protein specifically modulating one pathway?
3. While recent work has begun to identify viral proteins that alter (mostly) carbon metabolism, detailed mechanistic insights remain rare. What are the molecular events that lead to the upregulation of a metabolic pathway during viral infection?
4. The field of viral metabolism is in its infancy, and research has mostly focused on central carbon metabolism and lipid metabolism. Do viral proteins specifically alter lesser-studied pathways, such as purine/pyrimidine metabolism, or non-essential or non-proteinogenic amino acid metabolism?
5. Does pharmacologic targeting of metabolism provide a therapeutic opportunity for enhancing the activity of direct-acting antivirals for successfully treating viral infections?
6. Beyond regulating lipogenic transcription factors, how are lipid metabolic pathways differentially regulated by a virus?
7. Does a virus protein alter both lipid synthesis and the metabolic pathways that make the metabolites that feed into lipids, including acetyl-CoA, DHAP, serine, choline, and others?
8. Do different cell types in the infected environment alter the metabolic interplay between cells, and do viral proteins regulate these pathways?
9. What is the connection between the kinetics of virus replication and metabolism? How do viral proteins ensure that metabolites and lipids are present when the virus needs them?
